# Supplementary material for: The causal role of circulating inflammatory markers in osteoporosis: a bidirectional Mendelian randomized study
Source: Front Immunol. 2024 Jul 18;15:1412298. doi: 10.3389/fimmu.2024.1412298 (PMC11291241; doi:10.3389/fimmu.2024.1412298)
Supplement: Supplementary file 1 [file DataSheet_1.zip › supplementary material/CIM-OSTEOPOROSIS /GCST90274824.csv_leave_one_out.pdf]

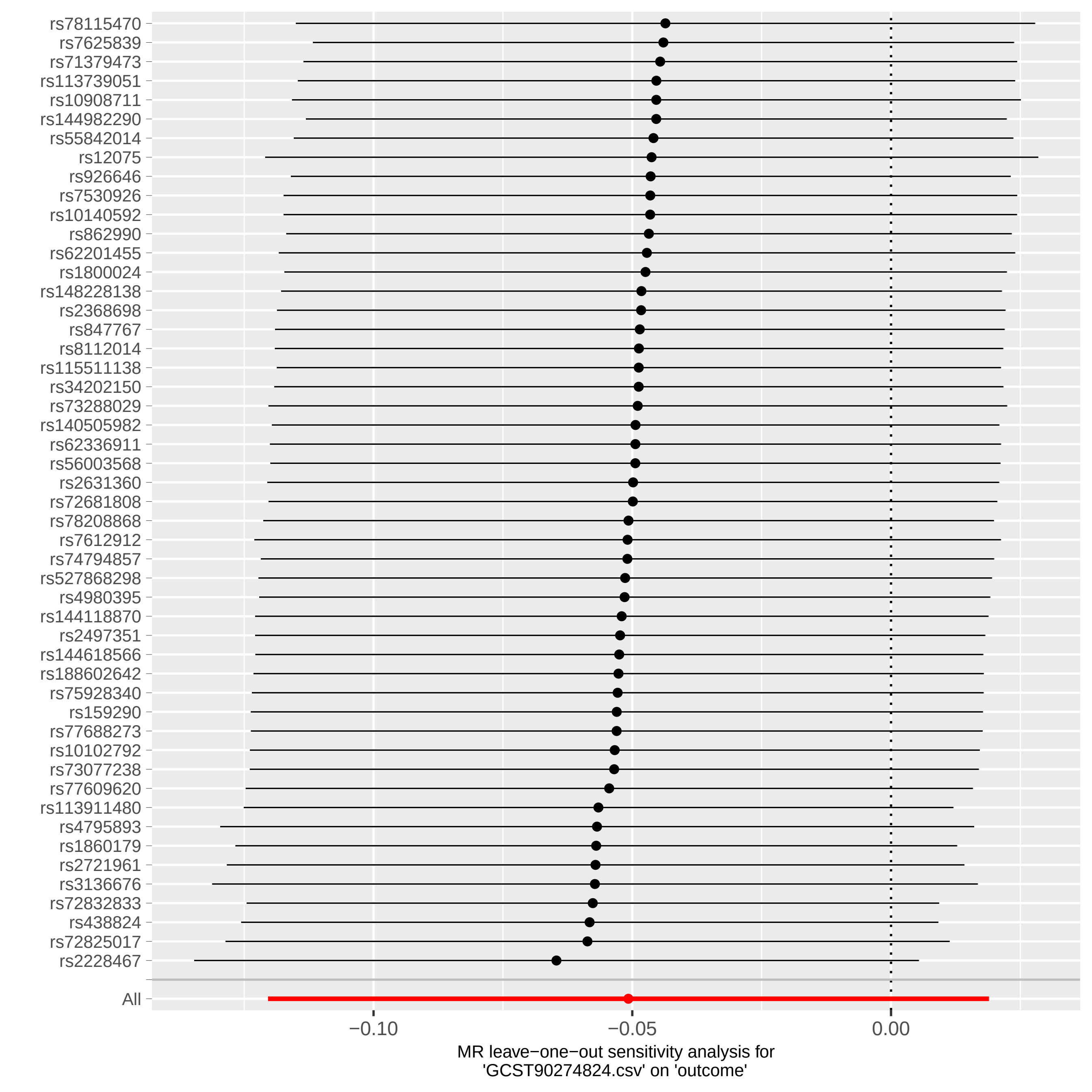
rs78115470
rs7625839
rs71379473
rs113739051
rs10908711
rs144982290
rs55842014
rs12075
rs926646
rs7530926
rs10140592
rs862990
rs62201455
rs1800024
rs148228138
rs2368698
rs847767
rs8112014
rs115511138
rs34202150
rs73288029
rs140505982
rs62336911
rs56003568
rs2631360
rs72681808
rs78208868
rs7612912
rs74794857
rs527868298
rs4980395
rs144118870
rs2497351
rs144618566
rs188602642
rs75928340
rs159290
rs77688273
rs10102792
rs73077238
rs77609620
rs113911480
rs4795893
rs1860179
rs2721961
rs3136676
rs72832833
rs438824
rs72825017
rs2228467
All
−0.10
−0.05
0.00
MR leave−one−out sensitivity analysis for
'GCST90274824.csv' on 'outcome'
